# Chromosomal divergence and evolutionary inferences in Pentatomomorpha infraorder (Hemiptera, Heteroptera) based on the chromosomal location of ribosomal genes

**DOI:** 10.1371/journal.pone.0228631

**Published:** 2020-02-04

**Authors:** Tatiani Seni de Souza-Firmino, Kaio Cesar Chaboli Alevi, Mary Massumi Itoyama

**Affiliations:** 1 Departamento de Biologia, Instituto de Biociências, Letras e Ciências Exatas, Universidade Estadual Paulista “Júlio de Mesquita Filho”, Câmpus de São José do Rio Preto, São José do Rio Preto, SP, Brazil; 2 Departamento de Ciências Biológicas, Faculdade de Ciências Farmacêuticas de Araraquara, Universidade Estadual Paulista “Júlio de Mesquita Filho”, Câmpus de Araraquara, Araraquara, SP, Brazil; Texas A&M University College of Veterinary Medicine, UNITED STATES

## Abstract

With the objective of assisting in the understanding of the chromosome evolution of Pentatomomorpha and in the quest to understand how the genome organizes/reorganizes for the chromosomal position of the 45S rDNA in this infraorder, we analyzed 15 species (it has being 12 never studied before by FISH) of Pentatomomorpha with the probe of 18S rDNA. The mapping of the 45S gene in the Coreidae family demonstrated that the species presented markings on the autosomes, with the exception of *Acanthocephala parensis* and *Leptoglossus gonagra* that showed markers on m-chromosomes. Most species of the Pentatomidae family showed marking in the autosomes, except for two species that had 45S rDNA on X sex chromosome (*Odmalea* sp. and *Graphosoma lineatum*) and two that showed marking on the X and Y sex chromosomes. Species of the Pyrrhocoridae family showed 18S rDNA markers in autosomes, X chromosome as well as in Neo X. The Largidae and Scutelleridae families were represented by only one species that showed marking on the X sex chromosome and on a pair of autosomes, respectively. Based on this, we characterized the arrangement of 45S DNAr in the chromosomes of 12 new species of Heteroptera and discussed the main evolutionary events related to the genomic reorganization of these species during the events of chromosome and karyotype evolution in Pentatomomorpha infraorder.

## Introduction

The Heteroptera suborder (Insecta, Hemiptera) is the largest and most diverse group of insects with incomplete metamorphosis, being formed by seven infraorders (Leptopodomorpha, Gerromorpha, Nepomorpha, Pentatomomorpha, Cimicomorpha, Dipsocoromorpha and Enicocephalomorpha) composed of more than 40,000 species distributed in approximately 80 families [1, 2]. These insects of medical and agricultural importance have a great diversity of habitats (aquatic, terrestrial and parasitic of birds and bats) and they present different feeding habits, such as phytophagous, predators, necrophagous and hematophagous [1].

The Pentatomomorpha infraorder is the second largest and one of the most economically important of Heteroptera with approximately 15,000 species and 40 families worldwide [1]. This infraorder can cause great damage to agricultural production, and act on the transmission of phytopathogens [1]. However, not all species of Pentatomomorpha are harmful, because there are examples that act in the biological control of agricultural pests, as well as in environmental bioindication, increasing or decreasing the diversity of species when great environmental disturbances occur [1, 3].

The first cytogenetic survey in Heteroptera was initiated in 1891 with the description of the spermatogenesis of *Pyrrhocoris apterus* (Hemiptera, Pyrrhocoridae) [4]. Posteriorly, several chromosomal and karyotype analysis were performed, with emphasis on the characterization of the chromosome number, relative size of the autosomes and sex chromosomes and description of the sex-determination system [5–7]. These insects of medical and agricultural importance have unique chromosomal characteristics as holocentric chromosomes, micro chromosomes (m-chromosomes), which are generally aquiasmatic during the onset of prophase I, inverted meiosis for sex chromosomes and end to end pairing [5, 6, 8, 9].

The diploid number of chromosomes in Heteroptera varies from 2n = 4 [*Lethocerus* sp. (Hemiptera, Belostomatidae)] to 2n = 80 [(*Lopidea marginalis* (Hemiptera, Miridae)], with the majority of species from 12 to 34 chromosomes (70% of species), being the most common diploid number of 2n = 14 (460 species) [9]. Chromosomal sex-determination system mechanisms known are divided into simple [XY/XX (71.4%) and X0/XX (14.7%)] and multiples [X_**n**_Y/X_**n**_X_**n**_, X_**n**_0/X_**n**_X_**n**_ and XY_**n**_/XX (13.5%)] [6, 7]. In addition, there is the Neo XY system that basically is the fusion of sex chromosomes X or Y with autosomes [9].

It is suggested that the chromosomal sex-determination system simple and multiple with presence of the Y sex chromosome (XY and X_**n**_Y), present in Nepoidea and Gelastocoroidea superfamilies, is a plesiomorphic feature and the presence of a pair of m-chromosomes and different chromosomal sex-determination system with absence of Y sex chromosome (X0 and X_**1**_X_**2**_0), present in Naucoroidea and Notonectoidea superfamilies, is an apomorphic feature [10–12]. Cytogenetic analysis suggest that the absence of m-chromosomes and the presence of Y sex chromosomes could be considered plesiomorphic characters, because it is believed that the sex systems X0 and X_**1**_X_**2**_0 originated through the loss of the Y sex chromosome (aneuploidy), as well as loss of Y followed by fragmentation of the X chromosome, respectively [12]; those sex-determination systems together with the presence of a pair of m-chromosomes could be considered as derived characters [12].

Fusion (simploidy) and fission (agmatoploidy), together with aneuploidies, are the main mechanisms of evolution of the karyotype in Heteroptera [5, 6, 13, 14, 15], which support the probable sex systems X_**n**_Y, X0 and X_**1**_X_**2**_0, as well as Neo XY, from the simple sex determination system (XY) [13, 16], being all previously reported sex-determination system already notified for the Pentatomomorpha infraorder [6, 17].

In relation to the survey Heteroptera suborder, there are currently three hypotheses: Sherbakov and Popov [18] and Mahner [11] were congruent in the hypotheses proposals, they proposed that Nepomorpha is a sister group of the remainder of Heteroptera. The other two hypotheses correspond to Wheeler et al. [19] and Xie et al. [20] that demonstrated that all infraordens are monophyletic with a phylogenetic relationship between Enicocephalomorpha (Nepomorpha (Leptopodomorpha (Gerromorpha + Dipsocoromorpha) + (Cimicomorpha + Pentatomomorpha))) for Xie et al. [20] and Enicocephalomorpha (Dipsocoromorpha (Gerromorpha (Nepomorpha (Leptopodomorpha (Cimicomorpha + Pentatomomorpha)))) for Wheeler [19], resulting in new questions about the evolutionary relations of these infraordens [2].

Due to these inconsistencies, the use of complementary techniques such as classical and molecular cytogenetic analysis, can help to elucidate the relationships between species, contributing to phylogenetic, evolutionary and taxonomic studies [5,6, 12, 21–24]. The use of fluorescent in situ hybridization (FISH) allows mapping of specific DNA sequences in the chromosomes of the species [25]. The chromosome mapping has been usually performed in Heteroptera, being the study of the number and location of the 45S ribosomal genes the most frequent [22–24, 26, 27]. This gene generally appears as repeated sequences and grouped into particular chromosomes, especially at the ends of autosomes and/or sex chromosomes [22–24, 26–28]. In different groups of insects, such as Coleoptera, Diptera, Hymenoptera, Lepidoptera and Orthoptera, the distribution of 45S rDNA has been applied with the objective of assisting in phylogenetic, taxonomic and evolutionary studies [22–24, 26, 29–34].

For the chromosomal mapping of the 45S ribosomal gene in Heteroptera, the 18S rDNA probe has been widely used [22–23, 26, 28, 35, 36]. With the aim of assisting in the understanding of the chromosome evolution of Pentatomomorpha and in the quest to understand how the genome organizes/reorganizes for the chromosomal position of the 45S rDNA in this infraorder, we analyzed 15 species (it has being 12 never studied before by FISH) of Pentatomomorpha with the probe of 18S rDNA.

## Methods

### Animals

A total of 150 adult males of 15 species were analyzed: nine species of the Coreidae family (*Acanthocephala parensis*, *Anasa bellator*, *Spartocera fusca*, *S*. *batatas*, *Dallacoris pictus*, *D*. *obscura*, *Leptoglossus zonatus*, *Lucullia flavovittata* and *Phthia picta*), five species of Pentatomidae (*Edessa collaris*, *Loxa virescens*, *Mormidea v-luteum*, *Odmalea* sp. e *Thyanta perditor*) and one species of the Scutelleridae family (*Pachycoris torridus*). The insects were collected by active search in the Institute of Biosciences, Letters and Exact Sciences (IBILCE / UNESP), São José do Rio Preto, São Paulo, Brazil (geographical coordinates: Latitude: -20.802, Longitude: -49.3707 20º 49' 13” South, 49º 22 '47' 'West) during the period from March 2015 to May 2018.

### Chromosome preparations

The insects were dissected, the testes were removed and fixed in Carnoy's solution (absolute ethanol: acetic acid, 3: 1). The slides were prepared using a portion of the tests which was macerated in 50% acetic acid and then dried on a hot plate at 45–50° C.

### DNA isolation

The DNA was obtained from a sample of muscle tissue of the *M*. *v-luteum* species. The sample was digested with proteinase K for 3 h, was added phenol/Tris-HCl, pH 8.0, followed by centrifugation and washing with phenol/Tris-HCl, pH 8.0 and isoamyl alcohol-chloroform. After centrifugation, isoamyl alcohol-chloroform was added and the DNA was precipitated with ice-cold absolute ethanol for 12h at -20º C and eluted in Tris EDTA (TE; 1:10) + RNAse. The 18S rDNA probes were generated by Polymerase Chain Reaction (PCR), using the primers: Foward 5'-AACCTGGTTGATCCTGCCA-3 'and Reverse 5'-CTGAGATCCAACTACGAGCTT-3' [37]. The obtained fragments were purified and sequenced. The sequences were subjected to the BLAST nucleotide [38] to confirm the identity of these sequences.

### Fluorescence in situ hybridization (FISH)

FISH was performed according to Pinkel et al. [39], with modifications of Cabral-de-Mello et al. [40]: the 18S rDNA probe obtained from *M*. *v-luteum* was used in all insects analyzed. The DNA fragments were labeled with biotin-14-dUTP (Invitrogen) by PCR and the products visualized by 1% agarose gel electrophoresis to verify the amplification of the sequences. FISH signals were detected using alexa-flu-488 (Life Technologies) and the preparations were stained with 4 ', 6-diamidine-2'-phenylindole dihydrochloride (DAPI) and then assembled using Vecta shield (Vector). The preparations were observed using an Olympus BX61 Fluorescence microscope with DP70 refrigerated digital camera. Images were merged and optimized for brightness and contrast using Adobe Photoshop CS2 software.

## Results and discussion

We characterize the number and distribution of the 45S DNAr for 15 species (being 12 never before studied by FISH) belonging to three families of the Pentatomomorpha (nine species of Coreidae, five of Pentatomidae and one of Scutelleridae) (Table 1).

**Table 1:** Chromosomal complement, number of clusters and chromosomal pair where there was the labeling of 45 rDNA in the infraorder Pentatomomorpha. A: autosomes, X: X sex chromosome, X: Y sex chromosome.

Pentatomomorpha Infraorder	Karyotype	Number of Clusters	FISH (45S rDNA)	References
**Coreidae family**				
*Spartocera batatas*	2n = 23 (20 + 2m + X0)	2	A	Present study
*S*. *fusca*	2n = 23 (20 + 2m + X0)	2	A	[41], Present study
*Dallacoris pictus*	2n = 21 (18 + 2m + X0)	2	A	Present study
*D*. *obscura*	2n = 21 (18 + 2m + X0)	2	A	Present study
*Hypselonotus interruptus*	2n = 19 (16 + 2m + X0)	2	A	[26]
*H*. *fulvus*	2n = 19 (16 + 2m + X0)	2	A	[26]
*Anasa bellator*	2n = 21 (18 + 2m + X0)	2	A	Present study
*Zicca annulata*	2n = 23 (20 + 2m + X0)	2	A	[26]
*Z*. *nigropunctata*	2n = 23 (20 + 2 m +X0)	2	A	[27]
*Althos obscurator*	2n = 25 (22 + 2m + X0)	2	A	[26]
*Lucullia flavovittata*	2n = 21 (18 + 2m + X0)	2	A	Present study
*Acanthocephala parensis*	2n = 21 (18 + 2m + X0)	2	m-chromosome	Present study
*Leptoglossus gonagra*	2n = 21 (18 + 2m + X0)	2	m-chromosome	[26]
*L*. *zonatus*	2n = 21 (18 + 2m + X0)	2	A	[26], Present study
*L*. *neovexillatus*	2n = 21 (18 + 2m + X0)	2	A	[27]
*Anisoscelis foliaceus*	2n = 27 (24 + 2 m + X0)	2	A	[27]
*Holhymenia histrio*	2n = 27 (24 + 2m + X0)	2	A	[26]
*Chariesterus armatus*	2n = 25 (22 + 2m + X0)	2	A	[26]
*Phthia picta*	2n = 21 (18 + 2m + X0)	2	A	[26], Present study
*Athaumastus haematicus*	2n = 21 (18 + 2m + X0)	2	A	[26]
*Acanonicus hahni*	2n = 19 (18 + X0)	2	A	[26]
*Cebrenis* sp.	2n = 23 (20 + 2m + X0)	2	A	[26]
*Pachylis argentinus*	2n = 15 (12 + 2m + X0)	2	A	[42]
*Holhymenia rubiginosa*	2n = 27 (24 + 2m + X0)	-	A	[43]
*Camptischium clavipes*	2n = 21 (18 + 2m + X0)	2	A	[44]
*Machtima crucigera*	2n = 21 (18 + 2 m + X0)	2	A	[27]
**Pentatomidae family**				
*Loxa virescens*	2n = 14 (12 + XY)	2	A	Present study
*Mormidea v-luteum*	2n = 14 (12 + XY)	2	A	Present study
*M*. *notulifera*	2n = 14 (12 + XY)	2	A	[27]
*Arvelius albopunctatus*	2n = 14 (12 + XY)	2	A	[26]
*Thyanta perditor*	2n = 14 (12 + XY)	2	A	Present study
*Odmalea* sp.	2n = 14 (12 + XY)	2	X	Present study
*Antiteuchus tripterus*	2n = 14 (12 + XY)	2	A	[26]
*Euschistus cornutus*	2n = 14 (12 + XY)	2	A	[26]
*E*. *heros*	2n = 14 (12 + XY)	2	A	[26]
*Edessa collaris*	2n = 14 (12 + XY)	2	A	Present study
*E*. *rufomarginata*	2n = 14 (12 + XY)	2	A	[26]
*E*. *impura*	2n = 14 (12 + XY)	2	A	[26]
*E*. *meditabunda*	2n = 14 (12 + XY)	-	A	[26]
*Eurydema oleracea*	2n = 14 (12 + XY)	2	X and Y	[28]
*Graphosoma lineatum*	2n = 14 (12 + XY)	1	X	[28]
*Nezara viridula*	2n = 14 (12 + XY)	2	A	[42]
*Oebalus poecilus*	2n = 14 (12 + XY)	4	A, X and Y	[27]
*Proxysalbo punctulatus*	2n = 14 (12 + XY)	2	A	[27]
**Rhopalidae family**				
*Harmostes prolixus*	2n = 13 (10 + 2m + X0)	2	A	[26]
**Pyrrhocoridae family**				
*Dysdercus ruficollis*	2n = 13 (12 + X0)	2	A	[45]
*D*. *imitator*	2n = 13 (12 + X0)	2	A	[26]
*D*. *fulvoniger*	2n = 13 (12 + X0)	2	A	[26]
*D*. *albofasciatus*	2n = 12 (10 + Neo XY)	2	neo X	[45]
*D*. *chaquensis*	2n = 13 (12 + X0)	2	A	[45]
*Pyrrhocoris apterus*	2n = 23 (22 + X0)	2	X	[28]
*Oncopeltus femoralis*	2n = 18 (16 + XY)	-	A	[46]
*Ochrimnus sagax*	2n = 14 (12 + XY)	-	A	[46]
*Lygaeus peruvianus*	2n = 12 (10 + XY)	-	A	[46]
**Largidae family**				
*Euryophthalmus rufipennis*	2n = 13 (12 + X0)	1	X	[26]
**Lygaeidae family**				
*Oxycarenus lavaterae*	2n = 18 (14 + 2m + XY)	2	A	[28]
**Scutelleridae family**				
*Pachycoris torridus*	2n = 14 (12 + XY)	2	A	Present study

With regard to the diploid chromosome complements of the Pentatomomorpha infraorder, the representatives of the Coreidae family presented karyotype ranging from 2n = 15 to 27, with sex-determination system XX/XO and presence of m-chromosomes (except *Acanonicus hahni*) (Table 1). The Pentatomidae and Scutelleridae families had the same number of chromosomes, namely, 2n = 14 (12A + XY) and sex-determination system XX/XY (Table 1). Pyrrhocoridae presented a karyotype varying from 2n = 12 to 18, with chromosomal of sex-determination system XY, X0 and Neo XY (Table 1), the Rhopalidae, Largidae and Lygaeidae families presented sex-determination system X0, X0 and XY, respectively (Table 1), being detected m-chromosomes in Rhopalidae and Lygaeidae (Table 1).

**Fig 1:**
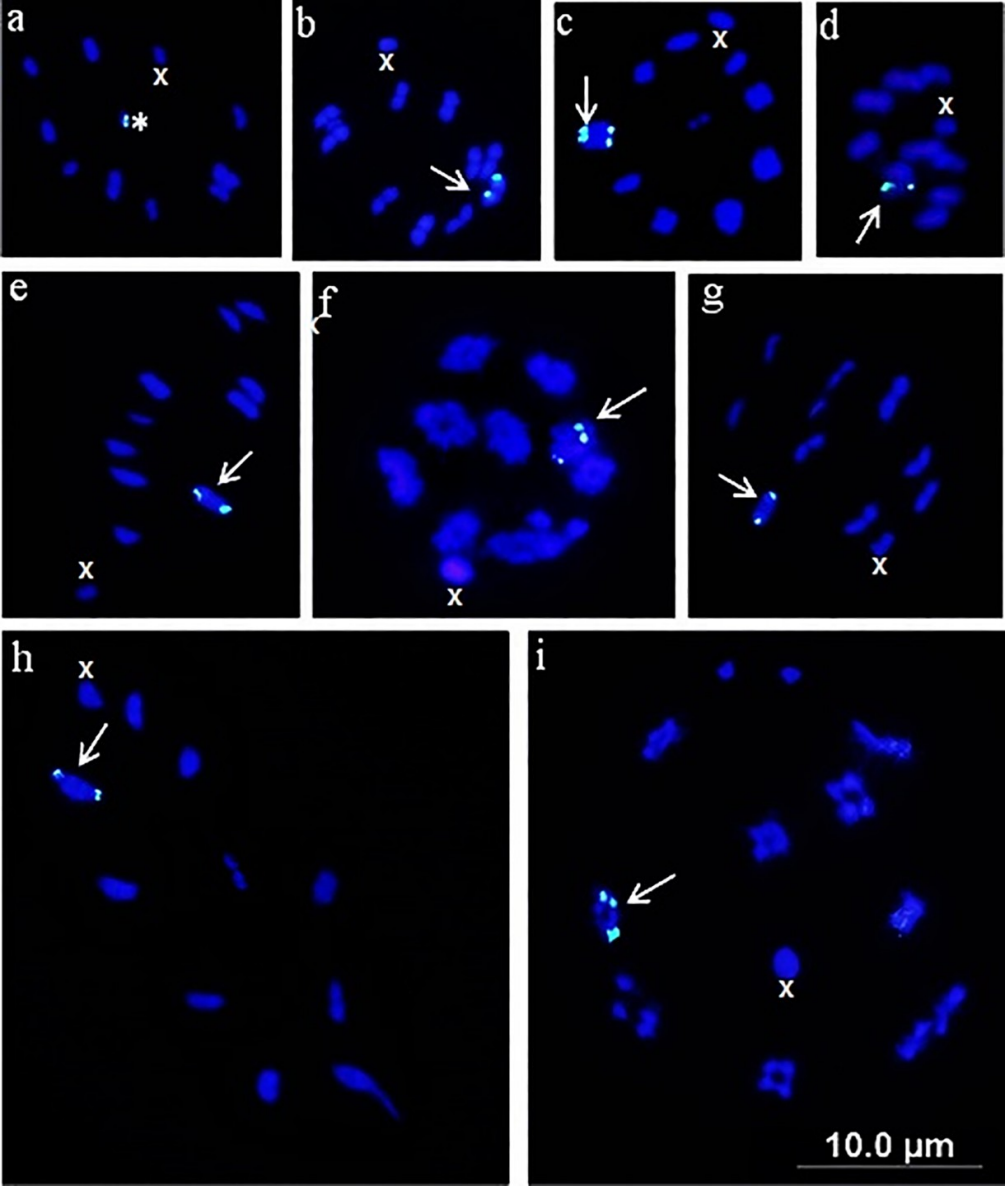
Fluorescent in situ hybridization using the 18S probe in species of the family Coreidae. a) *Acanthocephala parensis*, b) *Spartocera fusca*, c) *Phthia picta*, d) *Leptoglossus zonatus*, e) *Lucullia flavovittata*, f) *Dallacoris pictus*, g) *Anasa bellator*, h) *Spartocera batatas* and i) *D*. *obscura*. Asterisk: marking on the m-chromosome, Arrow: terminal marking on the autosomes, X: X sex chromosome, Y: Y sex chromosome. Bar: 10 μm.

The mapping of the 45S gene in the Coreidae family demonstrated that the species presented markings on the autosomes (Table 1, Fig 1B**–**1I), with the exception of *A*. *parensis* and *Leptoglossus gonagra* that showed markers on m-chromosomes (Table 1, Fig 1A). Taking into account that Coreidae is a monophyletic group [47] and that most species of this family share m-chromosomes, sex-determination system X0 and presence of the 45S gene in a pair of autosomes (Table 1), we suggest that the main events that led to numerical variation in the karyotype of these insects came from agmatoploidy/simploidy in the autosomes.

The FISH markings on m-chromosomes for *A*. *parensis* and *L*. *gonagra* (Table 1) when associated with the monophyly of the Coreidae family [47] allows us to propose that these chromosomes have an autosomal origin, since all other species of the Coreidae family showed marking in a pair of autosomes (Table 1) and agmatoploidy events are relatively common in holocentric chromosomes [15]. In addition, although Bressa et al. [48] emphasize that nothing can still be said about the information that m-chromosomes carry or what their function might be in the genetic system of the species that possess them, our results together with the results of Bardella et al. [26] demonstrate that these chromosomes have transcriptional activity (in this case, related to ribosomal biosynthesis by the presence of the 45S gene [49]), contributing, substantially, with the knowledge about these chromosomes little studied.

**Fig 2:**
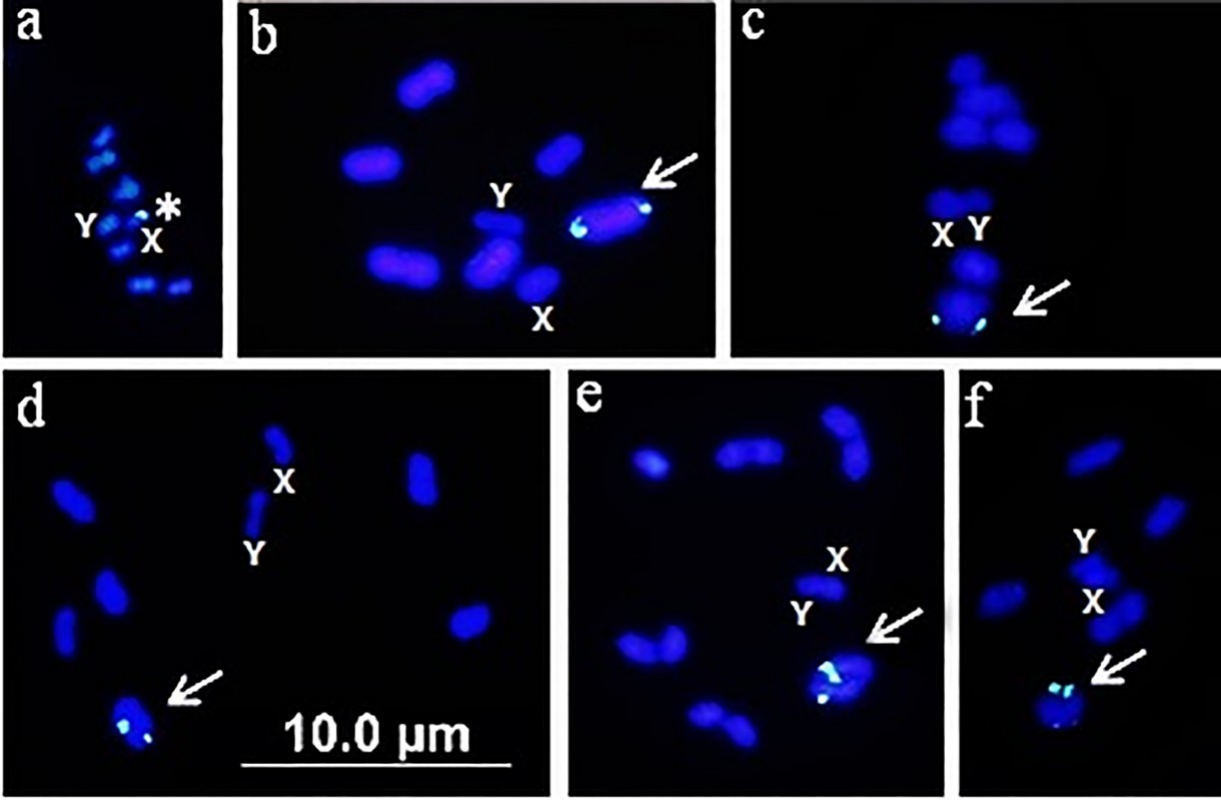
Fluorescent in situ hybridization using the 18S probe in species of the families Pentatomidae and Scutelleridae. a) *Odmalea* sp., b) *Mormidea v-luteum*, c) *Edessa collaris*, d) *Loxa virescens* e) *Thyanta perditor*, f) *Pachycoris torridus*. Asterisk: X sex chromosome, Arrow: terminal marking on the autosomes, X: X sex chromosome, Y: Y sex chromosome. Bar: 10 μm.

Most species of the Pentatomidae family showed marking in the autosomes (Table 1, Fig 2B**–**2E), except for two species that had 45S rDNA on X sex chromosome (*Odmalea* sp. and *Graphosoma lineatum*) (Table 1, Fig 2A), one that showed marking on the X and Y sex chromosomes (*Eurydema oleracea*) and one that showed marking on the autosome, as well as X and Y sex chromosomes (*Oebalus poecilus*) (Table 1). Rebagliati et al. [50] proposed that the maintenance of the chromosome number in Pentatomidae is associated with genomic stability. However, Bardella et al. [27] from the results of the rDNA mapping suggest that although the chromosome number is constant, different mechanisms of genomic reorganization are in place, causing amplification and dissemination of repetitive DNAs without the occurrence of macro-chromosomal alterations. Different from that observed for Coreidae, which justifies the variation of the locations of the ribosomal gene based on chromosomal breaks, on the basis of karyotypic stability (2n = 14) and the monophyletic origin of the Pentatomidae [51], we suggest that the mechanisms that led to the 45S gene diversification in this family are related to transposition elements (TEs), as suggested by Panzera et al. [22] and Pita et al. [23, 24] for the chromosome diversification of triatomines.

Species of the Pyrrhocoridae family showed 18S rDNA markers in autosomes, X chromosome as well as in Neo X (Table 1). Analyzing specifically the species of the *Dysdercus* genus, all species (except *Dysdercus albofasciatus*) present 2n = 13 (12 + X0) chromosomes and FISH labeling in a pair of autosomes (Fig 1). Based on the origin of the sex chromosomes NeoX and NeoY (from the fusion of the X or Y chromosome with an autosomes [9]) and on the karyotype and chromosomal homogeneity of the *Dysdercus* genus, Bressa et al. [45] suggest that the ancestral karyotype of *Dysdercus* is XO and that the neo-X chromosome evolved by insertion of the original X chromosome into one NOR-bearing autosome (and as a consequence, the homologue of this NOR-autosome became the neo-Y chromosome). These authors support this hypothesis based on four factors, namely, (i) reduced chromosome number by one pair when compared to other species of the genus, (ii) two heteropycnotic chromatin bodies in the diffuse stage, indicating separation of the ancestral X chromosome into two segments, (iii) occurrence of one or, less frequently, two terminal chiasmata in the neo-sex chromosome bivalent, and (iv) reduction segregation of the neo-X neo-Y bivalent at anaphase I.

However, taking in consideration that other species of the family Pyrrhocoridae have XY sex determination system (Table 1), we cannot rule out the hypothesis that possibly the ancestor of the species of *Dysdercus* genus may have presented a XY sex determination system and the chromosomal diversification could have derived in two different ways: i) an aneuploidy event occurred for the Y chromosome which resulted in the XO sex determination system and ii) a simplify event occurred between the XY sex chromosome pair and the pair of autosomes that had the 45S gene and subsequently there was loss of the ribosomal locus of the neo Y chromosome. The first hypothesis can be sustained by the simple fact that other species of the Pyrrhocoridae family present a sex-determination system XY (Table 1) and the second hypothesis can be based on the heterochromatic nature of the X sex chromosome of the insects of the Pentatomomorpha infraorder [6], because with the intention of minimizing the deleterious effects of TEs, they are often directed to regions of heterochromatin [52, 53]. This same hypothesis of transfer of 45S rDNA by TEs and subsequent loss of the ribosomal gene was used by Pita et al. [24] to explain the diversification of ribosomal genes in triatomines of the Rhodniini tribe.

The Largidae and Scutelleridae families were represented by only one species that showed marking on the X sex chromosome (Table 1) and on a pair of autosomes (Table 1, Fig 2F), respectively. Although they are initial characteristics important and shared with other families of the Pentatomomorpha infraorder (Table 1), new species should be analyzed for evolutionary inferences to be made.

## Conclusions

Based on this, we characterized the arrangement of 45S DNAr in the chromosomes of 12 new species of Heteroptera and discussed the main evolutionary events related to the genomic reorganization of these species during the events of chromosome and karyotype evolution in Pentatomomorpha infraorden.
